# Beyond serology: saccharide profiling enables identification of antigenically similar *Leptospira* and prompts re-evaluation of bacterial lipopolysaccharide evolution

**DOI:** 10.3389/fmolb.2025.1581587

**Published:** 2025-06-17

**Authors:** Aleksandra J. Lewicka, Jan J. Lyczakowski, Laura Pardyak, Klaudia Dubniewicz, Dariusz Latowski, Zbigniew Arent

**Affiliations:** ^1^ Department of Diagnostics and Clinical Sciences, Faculty of Veterinary Medicine, University of Agriculture in Krakow, Kraków, Poland; ^2^ Department of Plant Biotechnology, Faculty of Biochemistry, Biophysics and Biotechnology, Jagiellonian University, Kraków, Poland; ^3^ Department of Infectious Diseases and Public Health, Faculty of Veterinary Medicine, University of Agriculture in Krakow, Kraków, Poland; ^4^ Department of Plant Physiology and Biochemistry, Faculty of Biochemistry, Biophysics and Biotechnology, Jagiellonian University, Kraków, Poland

**Keywords:** lipopolysaccharide, leptospirosis and lipopolysaccharide, serotyping, glycosyl transferase, molecular evolution

## Abstract

Leptospirosis is a zoonotic infectious disease of growing importance in both human and veterinary medicine. Gram-negative spirochetes of *Leptospira* are traditionally classified into serovars based on their antigenic identity, which must be ascertained to design effective treatment procedures for humans and appropriate vaccination strategies in pets and livestock. Unfortunately, identifying *Leptospira* serovars is challenging and currently requires access to a wide panel of reference strains, animal-derived antisera, or monoclonal antibodies. Here, we describe a new method for the identification of *Leptospira* serovars that is based on monosaccharide composition analysis of the polysaccharide part of bacterial lipopolysaccharide (LPS) structures. Our approach requires no animal sacrifice and can be implemented in any laboratory equipped for chromatographic analysis. An LPS sugar fingerprint that is specific to each bacterial isolate that we studied can be generated. Importantly, sugar profiling of LPS enables distinguishing *Leptospira* serovars that are antigenically very similar. Using our new approach, we discover that the LPS structures of two cattle pathogens belonging to two different species: *Leptospira interrogans* and *Leptospira borgpetersenii*, and to one serovar: Hardjo, can be distinguished despite sharing major similarities. Through extensive phylogenetic analysis, we reveal which specific glycosyltransferases of the LPS biosynthesis *rfb* locus likely drove the emergence of these similarities and identify a single glycosyltransferase that might have contributed to the formation of saccharide differences in the LPS structure. Our findings have implications for future work on the evolution of bacterial polysaccharide synthesis and highlight the importance of preventing horizontal gene transfer between pathogenic bacteria.

## Introduction

*Leptospira* is a genus of Gram-negative spirochetes that includes pathogens with major clinical relevance in human and veterinary medicine. *Leptospira* are characterized by the presence of lipopolysaccharide (LPS) molecules that are antigenic but have low endotoxic activity in animals (Sun et al., 2020). For epidemiological purposes, in addition to standard genetic species, *Leptospira* are classified into serovars that have different host ranges and geographical distributions (Arent et al., 2022). Correct annotation of serovars not only enhances our understanding of the epidemiology of these infections but is also crucial for the selection of strains for diagnostic purposes and for their use as components in vaccine development. The LPS is thought to be the main determinant of the serovar identity, with different genetic species frequently belonging to the same serovars and showing similar clinical importance. Previous work has demonstrated that the majority of genes implicated in LPS biosynthesis, including glycosyltransferases (GTs) responsible for polysaccharide formation, are encoded on the *rfb* locus of *Leptospira* (Medeiros et al., 2022). Importantly, the locus has been subject to horizontal gene transfer (HGT) events between *Leptospira* species, which likely contributed to serovar evolution (de la Peña-Moctezuma et al., 1999; Haake et al., 2004; Nieves et al., 2022; Ca Ferreira et al., 2024).

Current estimates indicate that leptospirosis affects more than one million people annually, with 60,000 deaths reported because of the condition (Centre for Disease Control and Prevention, 2024). Recent reports point to the prevalence of antibiotic resistance in specific serovars, forcing modifications of prescribed treatment protocols (Pineda et al., 2024). The disease is of particular importance in veterinary medicine. In cattle farming, leptospirosis decreases animal health, productivity, and fertility, particularly in unvaccinated herds. Chronic infections caused by serovar Hardjo are of primary concern, as they result in long-term renal and reproductive colonization (Sohm et al., 2023). Current estimates indicate up to 84% loss in gross margin for farms where outbreaks are not controlled (Carvalho et al., 2024). Importantly, vaccines for specific *Leptospira* serovars show only limited cross-reactivity (Barazzone et al., 2022; Rinehart et al., 2012), which necessitates designing dedicated vaccination strategies based on the prevalence of specific serovars in the area of interest.

Correct characterization of *Leptospira* serovars present in clinical or environmental samples is crucially important, but all currently available methods for detecting *Leptospira* serovars have some limitations. The gold standard is the cross-agglutinin absorption test (CAAT), which is a laborious method requiring bacterial culturing and access to a panel of sera isolated from animals exposed to specific serovars (Pinto et al., 2022; Rajapakse et al., 2015). Importantly, the identification of selected *Leptospira* serovars can be significantly enhanced through the use of specific monoclonal antibodies (Arent et al., 2023). However, due to the challenges in obtaining monoclonal antibodies that target serovar-specific epitopes, such antibodies are not currently available for all known serovars.

Recently, much attention has been given to the development of strategies for serovar determination based on PCR. Some progress has been made when targeting genes of the *rfb* locus of the *Leptospira* genome with a complex combination of primer pairs (Wenderlein et al., 2024) or with in-depth analysis of RT-qPCR amplification curves (Waggoner et al., 2014; Esteves et al., 2018; Ahmed et al., 2020). However, these tools still need validation and improvement to provide confident annotation of serovars with clinical relevance.

Here, we present a new approach to the determination of *Leptospira* serovars that relies on the analysis of the monosaccharide composition of the bacterial LPS structures. The method utilizes high-performance liquid chromatography (HPLC) to generate an LPS sugar profile that can be considered a fingerprint of each *Leptospira* serovar and isolate. We apply our tool to discriminate exemplary serovars with major relevance for veterinary medicine, demonstrating that LPS composition in cattle pathogens belonging to one serovar, Hardjo, and to two species, *L. interrogans* and *L. borgpetersenii*, can be distinguished, despite high antigenic similarity, which classical serological methods cannot differentiate. Through molecular phylogenetic analysis, we demonstrate which specific GTs in the *rfb* locus of *Leptospira* are likely responsible for the biosynthesis of the similar and differentiating LPS structures in the two genetically distant cattle pathogens.

## Materials and methods

### LPS isolation and compositional analysis

Each bacterial strain (Supplementary Table S1) was initially assigned to serogroup and serovar by cross-agglutination using a microscopic agglutination test described previously (Dikken and Kmety, 1978). Bacterial isolates were grown in biological triplicate in a T80/40/LH culture medium (Ellis et al., 1985) at 29°C for 7–10 days, after which their LPS was isolated as previously described (Bonhomme and Werts, 2020). LPS composition was analyzed following TFA hydrolysis as detailed in Supplementary Protocol S1. A graphical summary of the LPS isolation and compositional analysis is shown in Supplementary Figure S1. Statistical analysis was performed in R.

### Bioinformatics and molecular phylogenetics

Sequences of 16S rRNA were obtained from Silva (Quast et al., 2013). Sequences of GT2, GT4, and GTnc enzymes were sourced from the Carbohydrate Active Enzymes (CAZY - http://www.cazy.org/) database (Drula et al., 2022). Molecular phylogenies were reconstructed with MEGA11 (Tamura et al., 2021) using the WAG algorithm, including the gamma distribution of rates. A dendroscope was used for visualization of the GT2 phylogeny (Huson and Scornavacca, 2012).

## Results

### LPS monosaccharide composition analysis is a powerful tool to discriminate even closely related *Leptospira* serovars

**FIGURE 1:**
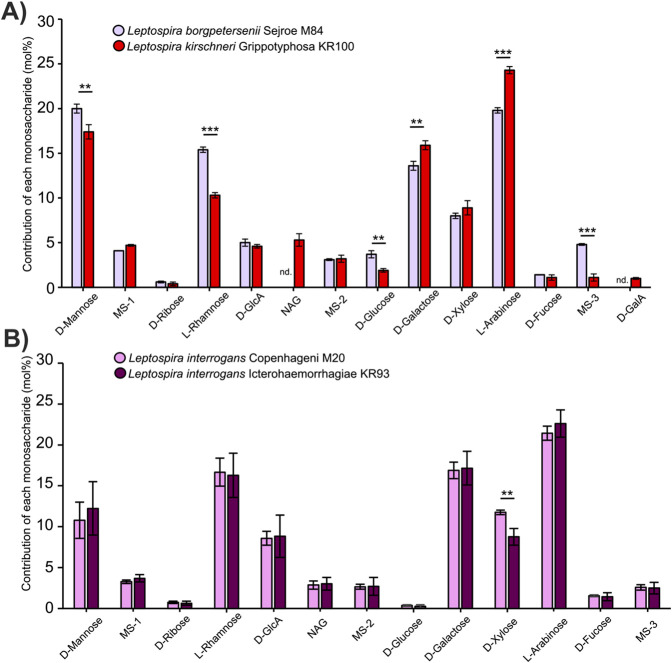
Monosaccharide composition of the LPS differs between *Leptospira* serogroups and serovars. **(A)** Monosaccharide composition of the LPS in *Leptospira borgpetersenii* serogroup Serjoe, serovar Serjoe, strain M 84, and in the *Leptospira kirschneri* serogroup Grippotyphosa, serovar Grippotyphosa, strain KR100. **(B)** Monosaccharide composition of the LPS in *Leptospira interrogans* serogroup Icterohaemorrhagiae, serovars: Icterohaemorrhagiae, strain KR93, and Copenhageni, strain M20. Error bars show the standard deviation of measurements performed for three biological replicates. Asterisks annotate significance in unpaired Student’s t-test with ** denoting p < 0.01 and *** denoting p < 0.001. Monosaccharide abbreviations used: NAG: N-acetylglucosamine; MS-1 to 3: unannotated monosaccharides with the same identity on all figures; D-GlcA: D-glucuronic acid; D-GalA: D-galacturonic acid. Nd. Indicates that a monosaccharide was not detected.

We hypothesized that the variable region of the LPS molecule can act as a unique molecular signature of individual *Leptospira* serovars. We hoped that the simplest possible approach, the analysis of LPS monosaccharide composition, would be sufficient to discriminate between serovars. To evaluate that, we first isolated LPS structures from two distant *Leptospira* strains: *L. borgpetersenii* serovar Serjoe, strain M84, and *Leptospira kirschneri* serovar Grippotyphosa, strain KR100. The strains belong to two different genetic species and two different serogroups, Serjoe and Grippotyphosa, respectively, making them a perfect case study to evaluate whether analysis of the monosaccharide composition of the LPS is a suitable method for serovar annotation. Following LPS breakdown to monosaccharides and HPLC, we quantified the molar contribution of each monosaccharide to the LPS molecule (Figure 1A). The analysis revealed major differences between the two isolates, including the presence or absence of different monosaccharides (N-acetyl-glucosamine and galacturonic acid) and quantitative differences in the content of six other monosaccharides. Encouraged by our initial findings, we wanted to establish if a more closely related pair of serovars could also be discriminated. To this end, we evaluated the LPS composition in *L. interrogans* sv. Copenhageni M20 and *L. interrogans* sv. Icterohaemorrhagiae KR93. These serovars are members of one serogroup (Icterohaemorrhagiae) and belong to one species, making them a challenging pair to distinguish even with well-established serological methods (Arent et al., 2023). We were encouraged by determining that the analysis of the monosaccharide composition of the LPS in the two serovars revealed quantitative differences in the amount of xylose (Figure 1B). This observation strongly suggests that our approach is capable of distinguishing even very closely related *Leptospira* serovars.

**FIGURE 2:**
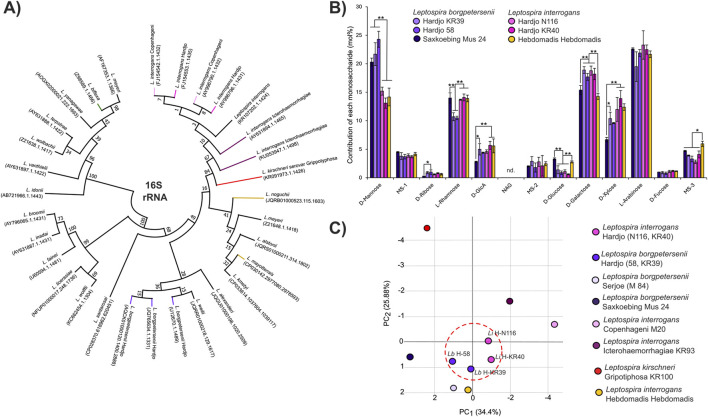
Composition of the LPS is similar in distant Leptospira species belonging to one serovar. **(A)** Result of maximum likelihood phylogenetic analysis of 16S rRNA sequence of *Leptospira,* including sequences from analyzed serovars. In total, 100 bootstrap replicates were used, and numerical confidence values are provided for all nodes. **(B)** Monosaccharide composition analysis of the LPS structures in *Leptospira borgpetersenii* serovar Hardjo (2 strains, KR39 and 58), *Leptospira interrogans* Hardjo (2 strains, N116 and KR40), *Leptospira interrogans* serogroup Hebdomadis serovar Hebdomadis, strain Hebdomadis, and in the *Leptospira borgpetersenii* serogroup Sejroe serovar Saxkoebing, strain Mus 24. Error bars show the standard deviation of measurements performed for three biological replicates. Asterisks annotate significance in ANOVA *post hoc* Tukey test with * denoting p < 0.05 and **p < 0.01. Nd. Indicates that a monosaccharide was not detected. **(C)** Principal component analysis (PCA) based on monosaccharide composition values (Supplementary Table S2) of all *Leptospira* serovars analyzed in this work. The LPS structures of strains belonging to the Hardjo serovar are similar and are marked with a dotted red circle.

Having established that our method allows confident discrimination between serovars, we set out to perform an analysis of LPS from two major cattle pathogens, both belonging to the serovar Hardjo but genetically classified into two different species: *L. interrogans* and *L. borgpetersenii*. Despite belonging to the same serovar and sharing cattle as the host species, the two are clearly members of distant clades when their 16S rRNA sequences are compared (Figure 2A). In line with their belonging to the same serovar, analysis of the LPS structures from isolates of *L. interrogans* sv. Hardjo and *L. borgpetersenii* sv. Hardjo revealed that the monosaccharide compositions of the variable region of their LPS structures are very similar (Figure 2B). Importantly, we were still able to discriminate the isolates from both species, mainly by looking at the mannose, rhamnose, and xylose contents in the LPS samples. To study the differences in the LPS monosaccharide composition, we performed a principal component analysis (PCA, Figure 2C) based on all our sugar fingerprint profiles. The PCA confirmed that the compositions of LPS in *L. borgpetersenii* sv. Hardjo and *L. interrogans* sv. Hardjo are more similar to one another than to that in other members of their respective genetic species.

### Neofunctionalization and horizontal transfer of specific glycosyltransferase genes in the *rfb* locus are the likely reasons for similarities in LPS structures

To understand the origin of the similarity in the LPS composition between the two cattle pathogens, we decided to focus our efforts on the study of GTs implicated in the formation of the glycan part of the LPS. We hypothesized that similarities in the GT toolbox of *L. borgpetersenii* sv. Hardjo and *L. interrogans* sv. Hardjo could indicate a shared biosynthetic pathway, likely originating from the previously reported horizontal gene transfer (HGT) events between the two species. The GT enzymes are subdivided into families, and the Carbohydrate Active Enzymes (CAZY) database can be used to identify families present in *Leptospira* genomes. The well-characterized *rfb* locus of *L. interrogans* serovar Hardjo strain Norma (Medeiros et al., 2022) comprises 12 GT enzymes that are categorized by CAZY. Of these, eight fall into the GT2 family, two into the GT4 family, and two are assigned to an as yet unknown family (GTnc). We decided to evaluate the similarities in these genes between Hardjo members from *L. interrogans* and *L. borgpetersenii* species.

**FIGURE 3:**
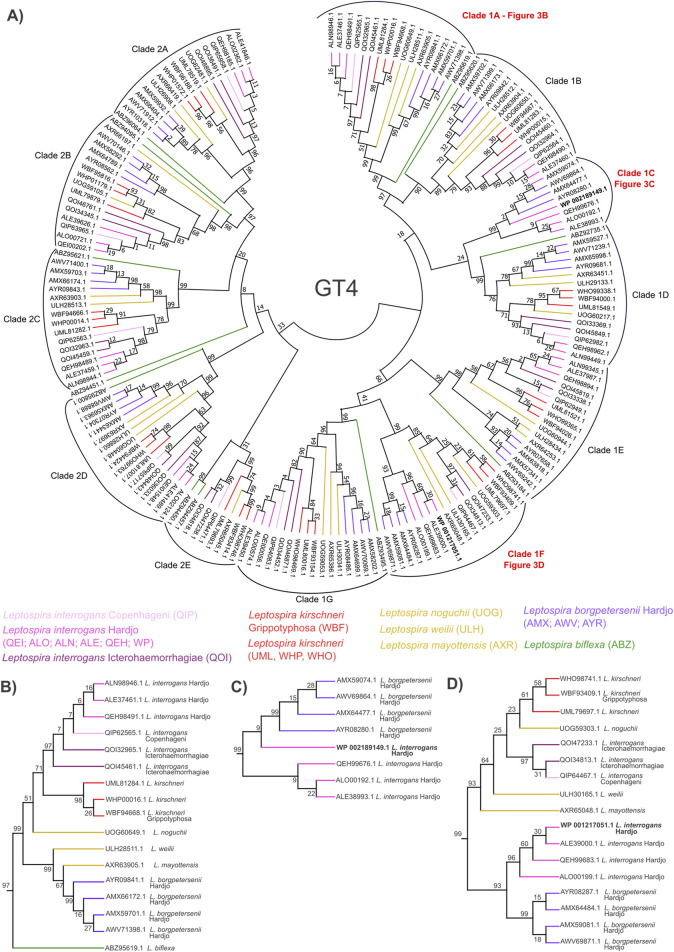
Phylogenetic analysis of *Leptospira* GT family 4. **(A)** Result of maximum likelihood phylogenetic analysis of *Leptospira* GT4 family protein sequences annotated by the CAZY database. One hundred bootstrap replicates were used, and numerical confidence values are provided for all nodes. Specific color codes are used for each serovar analyzed, with similar colors denoting members of one bacterial species. Letter codes are provided in brackets to indicate proteins from specific serovars. Sequences from the annotated *rfb* locus of *L. interrogans* are marked in red, and subtrees containing these sequences are presented on **(B–D)**, with **(B)** showing topology expected based on genetic similarities between *Leptospira* species, and panels **(C, D)** marking topologies indicating protein neofunctionalization and HGT events between *L. interrogans* sv. Hardjo and *L. borgpetersenii* sv. Hardjo.

To this end, we performed molecular phylogenetic analyses on the GT2 (Supplementary Figure S2), GT4 (Figure 3A), and GTnc (Supplementary Figure S3) sequences from a selection of available *Leptospira* genomes. For the analysis, we chose sequences from representatives of species and serovars for which we characterized the LPS composition and from species that are important for the separation of the *L. interrogans* and *L. borgpetersenii* clades (Supplementary Table S3; Figure 2A). We first evaluated GT4 enzymes (Figure 3A) and obtained a cladogram showing that Leptospiral family members separate into two major groups and a total of 12 distinct clades. Among these, most clades (10 of 12, example subtree in Figure 3B) showed topology akin to that observed in the 16S rRNA phylogeny, with *L. interrogans* sv. Hardjo and *L. borgpetersenii* sv. Hardjo sequences falling into two separate branches of a clade. Interestingly, for clades containing two GT4 enzymes from the *rfb* locus, the topology of the clade was different (Figures 3C,D). For one, the sequences were present only in members of the Hardjo serovar (Figure 3C), suggesting gene neofunctionalization prior to HGT. For the second one, the sequences of the Hardjo GT4 were more similar to one another than to those from other members of the same species (Figure 3D), marking the sequences as exchanged in an HGT event. A similar comparison was performed for GT2 (Supplementary Figure S2) and GTnc (Supplementary Figure S3) phylogenies, revealing neofunctionalization and HGT events for specific GT2 and GTnc enzymes.

## Discussion

In our work, we developed a new approach to the identification of *Leptospira* serovars that looks at the LPS monosaccharide compositions. We compared LPS structures both in distant serovars, belonging to different serogroups, such as Serjoe and Grippotyphosa (Figure 1A), and in much more similar ones, such as Copenhageni and Icterohaemorrhagiae, which belong to the same serogroup (Figure 1B). We observed clearly maintained trends in monosaccharide compositions of individual species and serovars (Supplementary Figure S4). For instance, serovar Hardjo is characterized by lower glucose and higher galactose content than other serovars. In all cases, we were able to distinguish individual *Leptospira* isolates based on the monosaccharide composition of their LPS structures. This confirmed that our method is suitable for discriminating *Leptospira* serovar pairs that are challenging to distinguish using standard serological approaches (Arent et al., 2023). Most critically, the comparative analysis of LPS structures with PCA allowed us to discriminate all studied isolates and group them according to serovar similarity. This opens the way for creating a reference library of LPS composition that can be used to assign *Leptospira* isolates to specific serovars.

Our method, unlike CAAT, does not require a ready collection of antisera nor any animal sacrifice (Pinto et al., 2022). Sugar fingerprinting relies on the direct analysis of the serovar-determining LPS antigen, which is advantageous when compared to PCR and RT-qPCR routes that attempt to define serovars indirectly. Moreover, our LPS compositional results are similar to those previously reported for two *Leptospira* isolates, which further validates our approach (Patra et al., 2015). Importantly, upon completion of the LPS compositional database, our tool will allow for rapid identification of novel *Leptospira* serovars, such as those isolated from unusual hosts (Grune et al., 2015). Sugar fingerprinting could be complemented by other ways of characterizing and classifying *Leptospira*, such as lipid A profiling (Pětrošová et al., 2023) or peptide analysis (Calderaro et al., 2014).

We applied our newly developed approach to extensively study the LPS structure in isolates belonging to two species: *L. interrogans* and *L. borgpetersenii*, and to one serovar, Hardjo, which are known cattle pathogens. We observed that their LPS structures are similar, which may be a result of the proposed HGT event between the two species (de la Peña-Moctezuma et al., 1999; Nieves et al., 2022). By combining the results from the performed phylogenies, we were able to identify six GTs that are shared between the *rfb* loci of *L. interrogans* and *L. borgpetersenii* sv. Hardjo (Table 1) and one GTnc (WP 000050431.1; ORF60) that is specific to *L. interrogans* sv. Hardjo only. We postulate that the shared enzymes, listed in Table 1, are likely responsible for the formation of a similar LPS antigen in Hardjo representatives adapted to cattle, and that the *L. interrogans*-specific GT may contribute to the formation of differences in LPS structure. What remains to be established is the exact activity of these enzymes and the advantage their function offers to cattle-adapted pathogens. In addition, such studies could be supplemented by linkage analysis, which would show how individual monosaccharide components of the LPS structures are connected and how LPS structure contributes to *Leptospira* antigenic identity. These questions are an active area of research for our group and must be addressed to fully understand the role of LPS during leptospirosis and in preventive vaccination.

**TABLE 1:** Summary of GT enzymes proposed to be implicated in the biosynthesis of the Hardjo-specific LPS.

Gene code in *L. interrogans* Hardjo strain Norma	ORF position in the *rfb* locus of *L. interrogans* Hardjo Norma	GT family	Specific evolutionary events concerning the gene
WP_002076509.1	ORF4	GT2	Neofunctionalization in the Hardjo ancestor, followed by an HGT event
WP_002189135.1	ORF44	GT2	Neofunctionalization in the Hardjo ancestor, followed by an HGT event
WP_002189149.1	ORF49	GT4	Neofunctionalization in the Hardjo ancestor, followed by an HGT event
WP_001217051.1	ORF56	GT4	HGT event in Hardjo, homologous GT present in other *Leptospira*
WP_000818302.1	ORF65	GT2	HGT event in Hardjo, homologous GT present in other *Leptospira*
WP_025177750.1	ORF66	GT2	HGT event in Hardjo, homologous GT present in other *Leptospira*

Our work describes a new powerful method for the identification of *Leptospira* serovars. Utilizing monosaccharide profiling, we are able to perform a detailed compositional analysis of the polysaccharide part of the LPS and to generate a unique sugar fingerprint of a serovar. This allows us to distinguish antigenically similar *Leptospira* and to start building a reference database of serovar-specific compositional profiles. In addition, through molecular phylogenetics, we identify the exact glycosyltransferase genes implicated in the HGT events that shaped the evolution of the LPS antigen. For that work, our efforts focus on *L. interrogans* sv. Hardjo and *L. borgpetersenii* sv. Hardjo. These two pathogens prevail in different parts of the globe but their isolates have been identified in overlapping areas, such as Europe and South America (van den Brink et al., 2023; Miller et al.,1991; Hagedoorn et al., 2024), where a shared host might have been a vessel for the horizontal gene exchange between bacterial species.

## Summary

Leptospirosis is a major disease of humans and animals caused by numerous distinct serovars of *Leptospira* bacteria. Determining which serovars are prevalent in a given area, or are causing the disease in a patient, is essential to design correct vaccination and treatment strategies. Currently we have a limited set of techniques available for identification of *Leptospira* serovars, with serological assays being the gold standard in the field. Unfortunately, these require the use of antisera, thus animal sacrifice, and often application of monoclonal antibodies. Our team has developed a new technique for determination of *Leptospira* serovar identity. By analysing the monosaccharide composition of leptospiral lipopolysaccharide (LPS) we were able to distinguish serovars showing very high degree of antigenic similarity. We further employed monosaccharide profiling to study selected serovars causing leptospirosis in cattle, and confirmed that their LPSs, though discernible with our method, share major structural similarities. Using molecular phylogenetic approaches we discover which exact glycosyltransferases may be implicated in the formation of these similar saccharide structures. Our work does not only provide a new tool for identification of *Leptospira*, but also sheds light on the evolution of bacterial polysaccharide biosynthesis, which may be relevant for our understanding of other host-pathogen interactions.

## Data Availability

The original contributions presented in the study are publicly available. This data can be found here: Mendeley Data, doi:10.17632/zf4scz7syj.1.
