# Supplementary material for: Beyond serology: saccharide profiling enables identification of antigenically similar Leptospira and prompts re-evaluation of bacterial lipopolysaccharide evolution
Source: Front Mol Biosci. 2025 Jun 17;12:1581587. doi: 10.3389/fmolb.2025.1581587 (PMC12208846; doi:10.3389/fmolb.2025.1581587)
Supplement: Supplementary file 1 [file Supplementaryfile1.docx]

**Supporting Information file for:**

Beyond Serology: Saccharide profiling enables identification of antigenically similar *Leptospira* and prompts re-evaluation of bacterial lipopolysaccharide evolution

**Authored by:**

Lewicka AJ^1,*^, Lyczakowski JJ^2^, Pardyak L^3^, Dubniewicz K^3^, Latowski D^4,*^, Arent Z^3^

^1.^ Department of Diagnostics and Clinical Sciences, Faculty of Veterinary Medicine, University of Agriculture in Krakow, Mickiewicza 21, 31-120, Kraków, Poland

^2.^ Department of Plant Biotechnology, Faculty of Biochemistry, Biophysics and Biotechnology, Jagiellonian University, Gronostajowa 7, 30-387 Kraków, Poland

^3.^ Department of Infectious Diseases and Public Health, Faculty of Veterinary Medicine, University of Agriculture in Krakow, Mickiewicza 21, 31-120, Kraków, Poland

^4.^ Department of Plant Physiology and Biochemistry, Faculty of Biochemistry, Biophysics and Biotechnology, Jagiellonian University, Gronostajowa 7, 30-387 Kraków, Poland

*Authors for correspondence: Aleksandra Lewicka (aleksandra.lewicka@urk.edu.pl) and Dariusz Latowski (dariusz.latowski@uj.edu.pl).

**Supporting Information Content:**

**Figure S1:** Method summary.

**Figure S2:** Result of maximum likelihood phylogenetic analysis of GT2 family members in selected *Leptospira* species including sequences from the annotated *rfb* locus of *L. interrogans* sv. Hardjo.

**Figure S3:** Phylogenetic analysis of the GTnc clade.

**Figure S4:** Trends in the monosaccharide composition of LPS in serogroups and genetic species included in the study.

**Table S1:** *Leptospira* reference strains used in the study

**Table S2:** Numerical values for monosaccharide percentage and standard deviation values reported in the manuscript.

**Table S3:** Sequences used for 16S rRNA and GT phylogenies.

**Protocol S1:** Detailed method for the analysis of monosaccharide composition of Leptospira LPS.

**
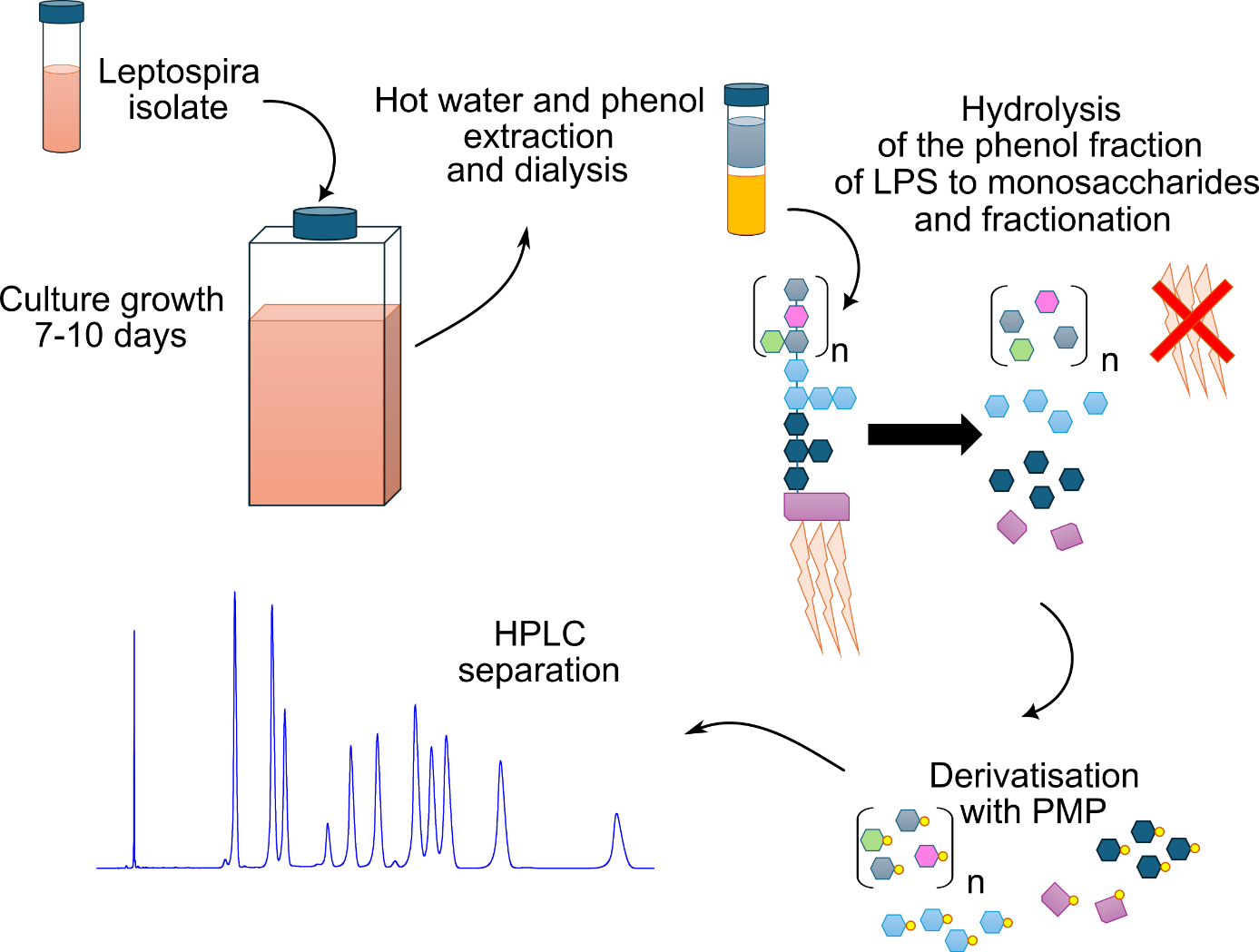
**

**Figure S1: Method summary.** Leptospira isolates are propagated into a larger volume culture (250 – 500 mL) and grown for 7-10 days in standardised conditions. Cell pellets are obtained and LPS is extracted by hot water and phenol extraction method as described by (Bonhomme and Werts, 2020). Phenol fraction of the LPS is further purified by dialysis and ultracentrifugation and used for monosaccharide composition analysis. LPS samples are hydrolysed into monosaccharides and chloroform-water separation is used to remove the lipid component of the LPS. Water-soluble monosaccharides are derivatised with PMP to allow absorbance-based detection. HPLC separation is performed to quantify the monosaccharide content. Monosaccharides, which form the variable region of the LPS (shown in square brackets) constitute the majority of the sugars in the LPS and hence are the ones detected in HPLC.


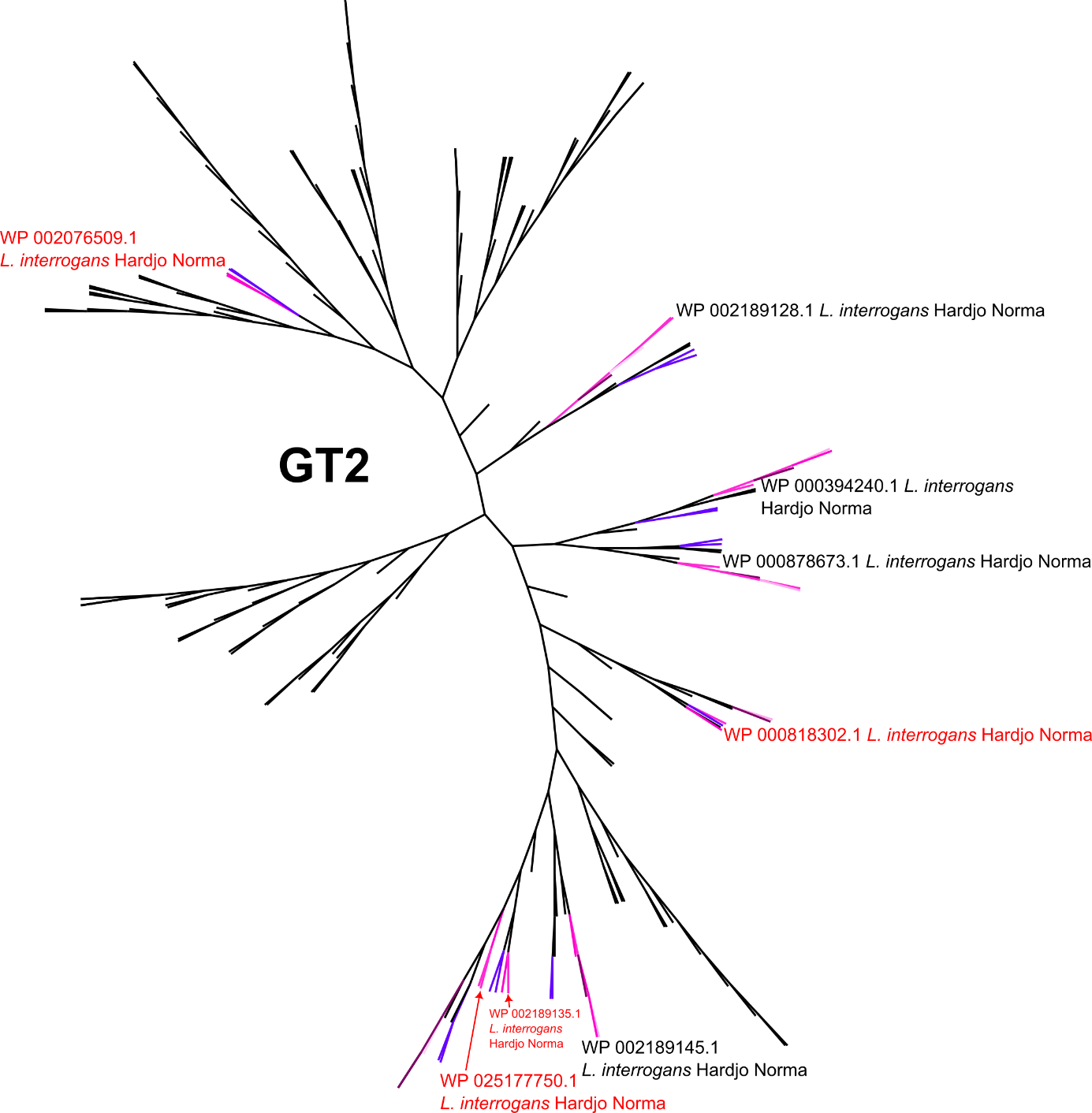


**Figure S2: Result of maximum likelihood phylogenetic analysis of GT2 family members in selected *Leptospira* species including sequences from the annotated *rfb* locus of *L. interrogans* sv. Hardjo.** Full sequences of all GT2 proteins analysed on this phylogeny and a Newick file of the phylogeny are available in a dataset associated with this publication (doi: 10.17632/zf4scz7syj.1). Branches corresponding to sequences from *rfb* locus of *L. interrogans* sv. Hardjo are highlighted in pink and labelled. Colour codes are provided to indicate if these sequences form one clade with sequences of *L. borgpetersenii* sv. Hardjo (marked in purple) or if they form one clade with these from genetically closely related serovars *L. interrogans* sv. Icterohaemorrhagiae and sv. Copenhageni (marked in dark and light pink respectively). *L. intterrogans* sv. Hardjo sequence codes that are closely related to these from *L. borgpetersenii* sv. Hardjo are written in red font.


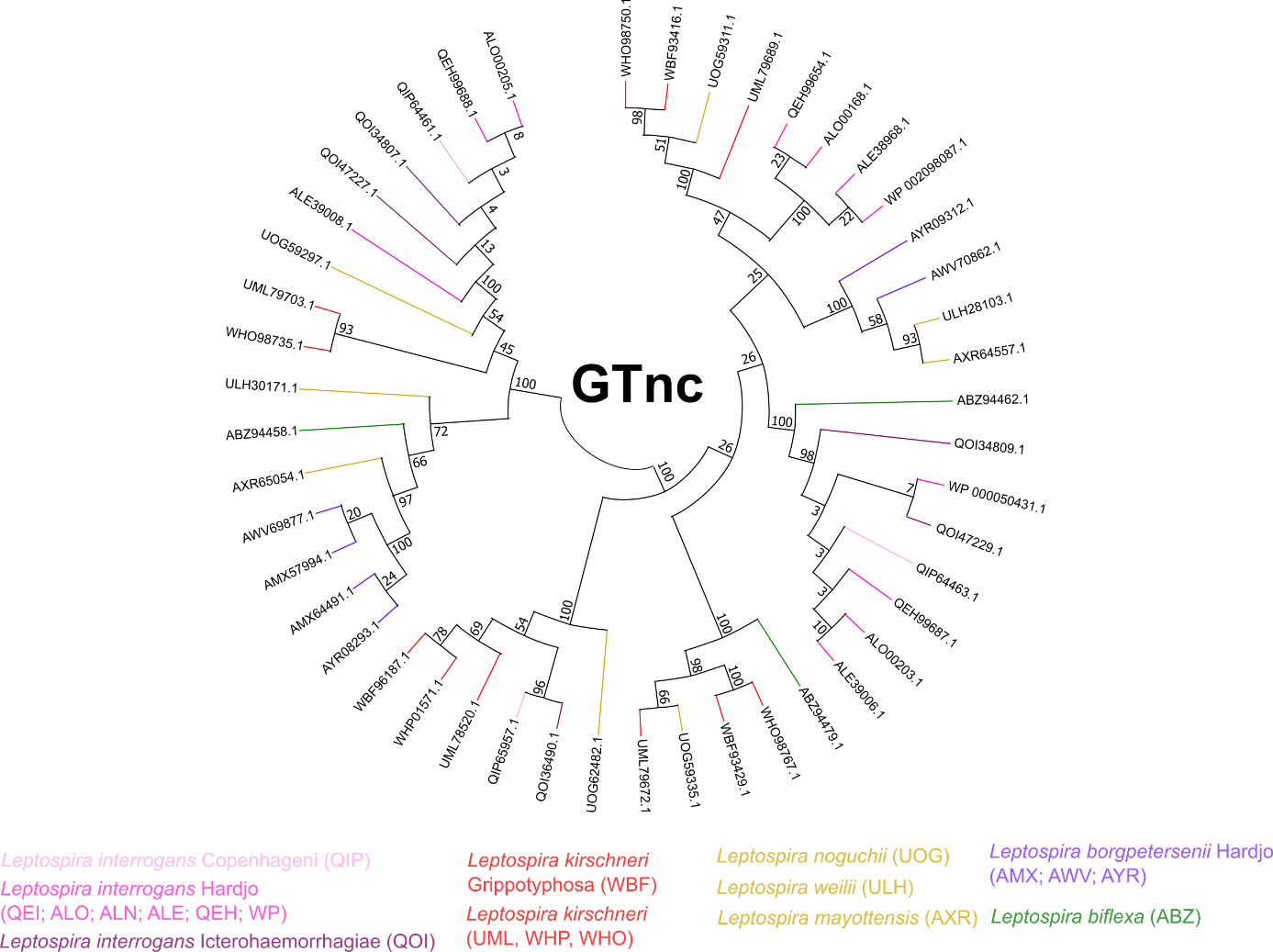


**Figure S3: Phylogenetic analysis of the GTnc clade.** Cladogram showing phylogenetic analysis of GTnc sequences from selected *Leptospira* isolates. Colour codes and letter codes specific to species, serovars and isolates are provided. Sequences from the characterised *rfb* locus of *L. interrogans* have a two-letter code WP.

**
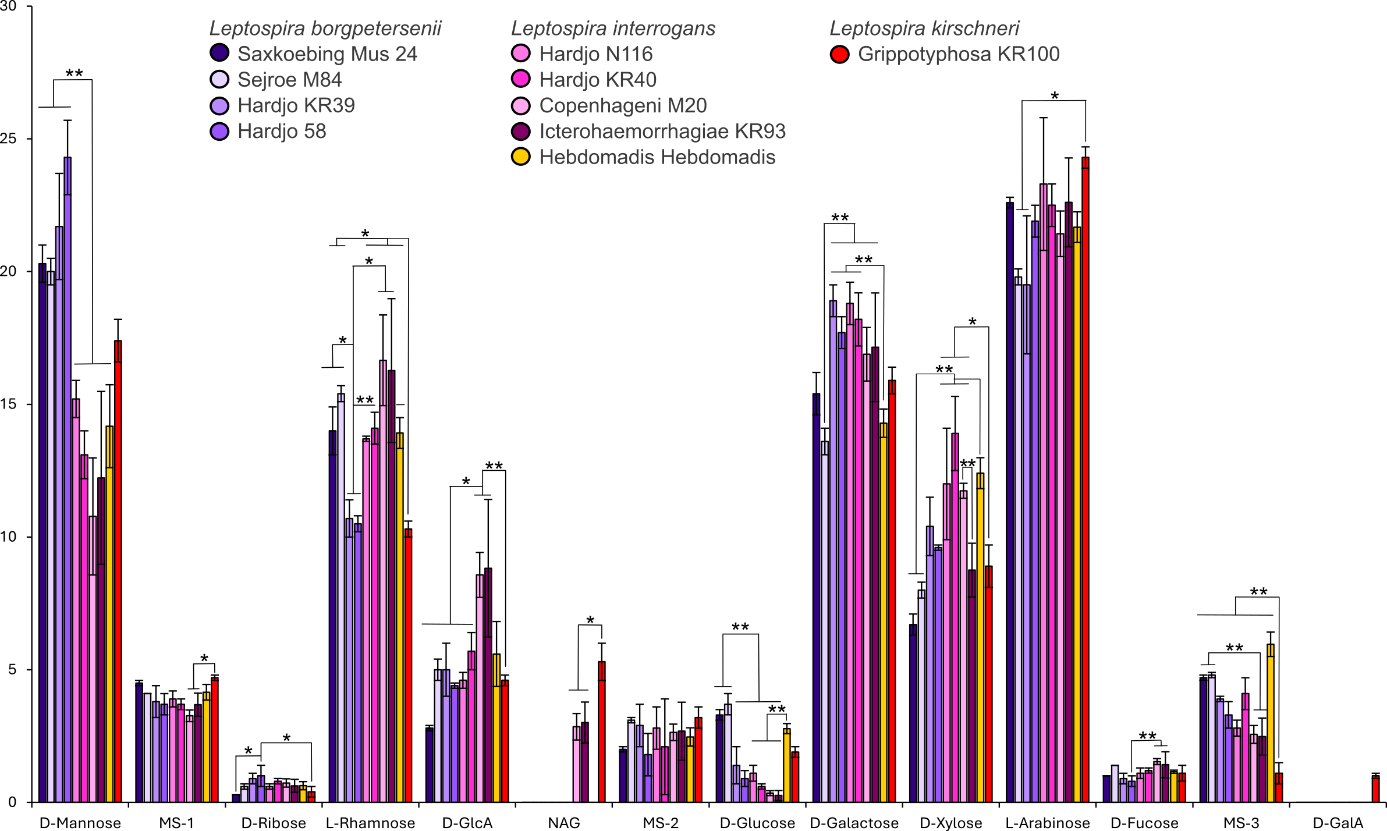
**

**Figure S4: Trends in the monosaccharide composition of LPS in serogroups and genetic species included in the study.** Samples from 10 bacterial strains, belonging to 3 genetic species, 4 overlapping serogroups and 7 serovars are shown. For each of the analysed samples, a unique sugar fingerprint of LPS that is specific and distinguishable from other isolates was obtained. Characteristic signatures of a given species (e.g. high mannose content in *L. borgpetersenii* and lower mannose content in *L. interrogans*) or a serovar (e.g. low glucose content and high galactose content in Hardjo serovar) are always maintained for each isolate. Error bars show standard deviation of measurements performed for three biological replicates. Asterisks annotate significance in ANOVA post-hoc Tukey test with * denoting p<0.05 and ** p<0.01.

**Table S1. *Leptospira* reference strains used in the study.**

| **Species** | **Serogroup** | **Serovar** | **Strain** |
| --- | --- | --- | --- |
| *L. interrogans* | Sejroe | Hardjo | KR40 |
| *L. interrogans* | Sejroe | Hardjo | N116 |
| *L. borgpeterseni* | Sejroe | Hardjo | KR39 |
| *L. borgpeterseni* | Sejroe | Hardjo | 58 |
| *L. interrogans* | Icterohaemorrhagiae | Icterohaemorrhagiae | KR93 |
| *L. borgpeterseni* | Sejroe | Sejroe | M84 |
| *L. interrogans* | Icterohaemorrhagiae | Copenhageni | M20 |
| *L. borgpeterseni* | Sejroe | Saxkoebing | MUS24 |
| *L. kirschneri* | Grippotyphosa | Grippotyphosa | KR100 |
| *L. interrogans* | Hebdomadis | Hebdomadis | Hebdomadis |

**Table S2** Numerical values for monosaccharide percentage and standard deviation (in brackets) values reported in the manuscript.

| Species, serovar, strain | D-Mannose | MS.1 | D- Ribose | L- Rhamnose | D- Gluc  uronic acid | NAG | MS.2 | D-Glucose | D- Galactose | D- Xylose | L- Arabinose | D- Fucose | MS.3 | D-Galact  uronic acid |
| --- | --- | --- | --- | --- | --- | --- | --- | --- | --- | --- | --- | --- | --- | --- |
| *L. borgpetersenii* Hardjo KR39 | 21.7  (2.0) | 3.8  (0.6) | 0.9  (0.2) | 10.7  (0.7) | 5.0  (1.0) | 0.0 | 2.9  (0.8) | 1.4  (0.7) | 18.9  (0.6) | 10.4  (1.1) | 19.5  (2.6) | 0.9  (0.2) | 3.9  (0.1) | 0.0 |
| *L. borgpetersenii* Hardjo 58 | 24.3  (1.4) | 3.7  (0.4) | 1.0  (0.4) | 10.5  (0.3) | 4.4  (0.1) | 0.0 | 1.8  (0.8) | 0.9  (0.3) | 17.7  (0.6) | 9.6  (0.1) | 21.9  (0.6) | 0.8  (0.2) | 3.3  (0.5) | 0.0 |
| *L. interrogans* Hardjo N116 | 15.2  (0.7) | 3.9  (0.3) | 0.6  (0.1) | 13.7  (0.1) | 4.6  (0.3) | 0.0 | 2.8  (0.8) | 1.1  (0.3) | 18.8  (0.8) | 12.0  (2.1) | 23.3  (2.5) | 1.1  (0.2) | 2.8  (0.3) | 0.0 |
| *L. interrogans* Hardjo KR40 | 13.1  (0.9) | 3.7  (0.2) | 0.8  (0.1) | 14.1  (0.6) | 5.7  (0.7) | 0.0 | 2.1  (1.8) | 0.6  (0.1) | 18.2  (1.0) | 13.9  (1.4) | 22.5  (0.8) | 1.2  (0.1) | 4.1  (0.6) | 0.0 |
| *L. interrogans* Copenhageni M20 | 10.8  (2.2) | 3.3  (0.2) | 0.7  (0.2) | 16.7  (1.7) | 8.6  (0.8) | 2.9  (0.5) | 2.6  (0.3) | 0.4  (0.1) | 16.9  (1.0) | 11.7  (0.3) | 21.4  (0.9) | 1.5  (0.1) | 2.6  (0.3) | 0.0 |
| *L. interrogans* Icterohaemorrhagiae KR93 | 12.2  (3.3) | 3.7  (0.4) | 0.6  (0.2) | 16.3  (2.7) | 8.8  (2.6) | 3.0  (0.8) | 2.7  (1.1) | 0.3  (0.2) | 17.1  (2.0) | 8.8  (1.0) | 22.6  (1.7) | 1.4  (0.5) | 2.5  (0.7) | 0.0 |
| *L. borgpetersenii* Saxkoebing Mus24 | 20.3  (0.7) | 4.5  (0.1) | 0.3  (0.0) | 14.0  (0.9) | 2.8  (0.1) | 0.0 | 2.0  (0.1) | 3.3  (0.2) | 15.4  (0.8) | 6.7  (0.4) | 22.6  (0.2) | 1.0  (0.0) | 4.7  (0.1) | 0.0 |
| *L. borgpetersenii* Sejroe M84 | 20.0  (0.5) | 4.1  (0.0) | 0.6  (0.1) | 15.4  (0.3) | 5.0  (0.4) | 0.0 | 3.1  (0.1) | 3.7  (0.4) | 13.6  (0.5) | 8.0  (0.3) | 19.8  (0.3) | 1.4  (0.0) | 4.8  (0.1) | 0.0 |
| *L. kirschneri* Grippotyphosa KR100 | 17.4  (0.8) | 4.7  (0.1) | 0.4  (0.2) | 10.3  (0.3) | 4.6  (0.2) | 5.3  (0.7) | 3.2  (0.4) | 1.9  (0.2) | 15.9  (0.5) | 8.9  (0.8) | 24.3  (0.4) | 1.1  (0.3) | 1.1  (0.4) | 1.0  (0.1) |
| *L. interrogans* Hebdomadis Hebdomadis | 14.2  (1.6) | 4.2  (0.3) | 0.6  (0.2) | 13.9  (0.6) | 5.6  (1.2) | 0.0 | 2.5  (0.3) | 2.8  (0.2) | 14.3  (0.5) | 12.4  (0.6) | 21.7  (0.6) | 1.2  (0.1) | 6.0  (0.5) | 0.0 |

**Table S3. Species and serovars analysed in 16S rRNA and GT phylogenies.**

| **Species, serovar (s. provided if detailed in database) and isolate** | **Species/sequence code and database** | **Phylogeny in which species/sequence was used** |
| --- | --- | --- |
| *L. alexanderi* | JQGU01000198 (Silva) | 16S rRNA |
| *L. alstonii* | JQRS01000211 (Silva) | 16S rRNA |
| *L. biflexa* | Z98589 (Silva) | 16S rRNA |
| *L. borgpetersenii* s. Hardjo | JQ765634 (Silva) | 16S rRNA |
| *L. borgpetersenii* s. Hardjo | U12670 (Silva) | 16S rRNA |
| *L. borgpetersenii* s. Hardjo | AQCK01000120 (Silva) | 16S rRNA |
| *L. broomii* | AY796065 (Silva) | 16S rRNA |
| *L. fainei* | U60594 (Silva) | 16S rRNA |
| *L. idonii* | AB721966 (Silva) | 16S rRNA |
| *L. inadai s. Kaup* | AY631887 (Silva) | 16S rRNA |
| *L. interrogans* s. Copenhageni | FJ154542 (Silva) | 16S rRNA |
| *L. interrogans* s. Copenhageni | AY996790 (Silva) | 16S rRNA |
| *L. interrogans* s. Hardjo | FJ154553 (Silva) | 16S rRNA |
| *L. interrogans* s. Hardjo | AY996796 (Silva) | 16S rRNA |
| *L. interrogans* s. Icterohaemorrhagiae | AY631894 (Silva) | 16S rRNA |
| *L. interrogans* s. Icterohaemorrhagiae | KU053947 (Silva) | 16S rRNA |
| *L. interrogans* s. Saxkoebing | KR107202 (Silva) | 16S rRNA |
| *L. kirschneri* s. Grippotyphosa | KR091973 (Silva) | 16S rRNA |
| *L. kmetyi* | CP033614 (Silva) | 16S rRNA |
| *L. licerasiae* | NFUP01000017 (Silva) | 16S rRNA |
| *L. mayottensis* | CP030142 (Silva) | 16S rRNA |
| *L. meyeri* | Z21648 (Silva) | 16S rRNA |
| *L. meyeri* | AF167353 (Silva) | 16S rRNA |
| *L. noguchii* | JQRB01000523 (Silva) | 16S rRNA |
| *L. santarosai* | CP028370 (Silva) | 16S rRNA |
| *L. terpstrae* | AY63188 (Silva) | 16S rRNA |
| *L. vanthielii* | AY631897 (Silva) | 16S rRNA |
| *L. weilii* | JQRR01000218 (Silva) | 16S rRNA |
| *L. wolbachii* | Z21638 (Silva) | 16S rRNA |
| *L. wolffii* | KC662454 (Silva) | 16S rRNA |
| *L. yanagawae* | AOGX02000021 (Silva) | 16S rRNA |
| *L. biflexa* s. Patoc Ames | 355278, ABZ (CAZY) | GT2, GT4, GTnc |
| *L. borgpetersenii* s. Hardjo 203 | 328971, AWV (CAZY) | GT2, GT4, GTnc |
| *L. borgpetersenii* s. Hardjo BK-30 | 328971, AMX6… (CAZY) | GT4, GTnc |
| *L. borgpetersenii* s. Hardjo BK-6 | 328971, AMX5… (CAZY) | GT2, GT4, GTnc |
| *L. borgpetersenii* s. Hardjo L49 | 328971, AYR (CAZY) | GT4, GTnc |
| *L. interrogans* s. Copenhageni SK1 | 44275, QIP (CAZY) | GT2, GT4, GTnc |
| *L. interrogans* s. Hardjo Hardjoprajitno | 38347, ALO (CAZY) | GT2, GT4, GTnc |
| *L. interrogans* s. Hardjo L53 | 176, QEI (CAZY) | GT4, GTnc |
| *L. interrogans* s. Hardjo Norma | 1279460, ALE (CAZY) | GT4, GTnc |
| *L. interrogans* s. Hardjo Norma | WP codes (Genebank) | GT2, GT4, GTnc |
| *L. interrogans* s. Icterohaemorrhagiae 898 | 90062, QOI4… (CAZY) | GT4, GTnc |
| *L. interrogans* s. Icterohaemorrhagiae Langkawi | 90062, QOI3… (CAZY) | GT2, GT4, GTnc |
| *L. kirschneri* 804Khv | 29507, WHO, WHP (CAZY) | GT2, GT4, GTnc |
| *L. kirschneri* FMAS PN-5 | 29507, UML (CAZY) | GT4, GTnc |
| *L. kirschneri* s. Grippotyphosa RedPanda1 | 29507, WBF (CAZY) | GT2, GT4, GTnc |
| *L. mayottensis* MDI222 | 1137606, AXR (CAZY) | GT2, GT4, GTnc |
| *L. noguchii* 201601331 | 28182, UOG (CAZY) | GT4, GTnc |
| *L. weilii* FMAS_PD2 | 28184, ULH (CAZY) | GT2, GT4, GTnc |

**Protocol S1: Method for the analysis of monosaccharide composition of *Leptospira* LPS.**

1. Phenol fraction of the LPS (0.5 - 2 mg) is incubated in 300 - 600 µL of 2M TFA for 1.5 - 2h at 120℃ to release monosaccharides from the polysaccharide part of the molecule.

2. The hydrolysate is cooled on ice, centrifuged and the supernatant is transferred to another tube and mixed with same volume of chloroform.

3. The chloroform:hydrolysate mix is strongly vortexed and spun for 1 minute. Top, aqueous, fraction is collected, transferred to another tube and desiccated under stream of nitrogen gas for 2-6h, until completely dry.

4. Monosaccharides, contained in the dried pellet, are derivatised with 3-methyl-1-phenyl-2-pyrazoline-5-one (PMP) through incubation for 2h at 70℃  in a mixture containing 25 µL 0.5 M PMP, 15 µL 0.5 M NaOH and 10 µL water.

5. Sample is neutralised by addition of 20 µL 0.5 M HCl and mixed with 600-1000 µL chloroform. The mix is heavily vortexed, spun at 1000 g for 1 minute and top aqueous layer is collected. The chloroform wash step is repeated and the final aqueous layer is transferred to a new tube and desiccated in vacuo.

6.  Dried sample is resuspended in 150 - 350  µL HPLC-grade water, filtered through nylon centrifugal filters and loaded onto HPLC.

7. HPLC separation is performed on Synergi Fusion-RP column (Phenomenex,  00G-4424-E0) through which 0.1 M phosphate buffer (pH in range 6.8 to 6.5) mixed with acetonitrile (82:18 v:v) is flown at 1 mL/min. Injection volume of 50 - 100  µL is used and sugar annotation is performed by analysis of elution profiles of monosaccharide standards labelled with PMP according to method described above (10 µL of 10 mM monosaccharide solution used instead of water in point 4 above).
